# Not your Mother’s MAPKs: Apicomplexan MAPK function in daughter cell budding

**DOI:** 10.1371/journal.ppat.1010849

**Published:** 2022-10-13

**Authors:** William J. O’Shaughnessy, Pravin S. Dewangan, E. Ariana Paiz, Michael L. Reese

**Affiliations:** 1 Department of Pharmacology, University of Texas, Southwestern Medical Center, Dallas, Texas, United States of America; 2 Department of Biochemistry, University of Texas, Southwestern Medical Center, Dallas, Texas, United States of America; Joan and Sanford I Weill Medical College of Cornell University, UNITED STATES

## Abstract

Reversible phosphorylation by protein kinases is one of the core mechanisms by which biological signals are propagated and processed. Mitogen-activated protein kinases, or MAPKs, are conserved throughout eukaryotes where they regulate cell cycle, development, and stress response. Here, we review advances in our understanding of the function and biochemistry of MAPK signaling in apicomplexan parasites. As expected for well-conserved signaling modules, MAPKs have been found to have multiple essential roles regulating both *Toxoplasma* tachyzoite replication and sexual differentiation in *Plasmodium*. However, apicomplexan MAPK signaling is notable for the lack of the canonical kinase cascade that normally regulates the networks, and therefore must be regulated by a distinct mechanism. We highlight what few regulatory relationships have been established to date, and discuss the challenges to the field in elucidating the complete MAPK signaling networks in these parasites.

## Introduction

**Fig 1:**
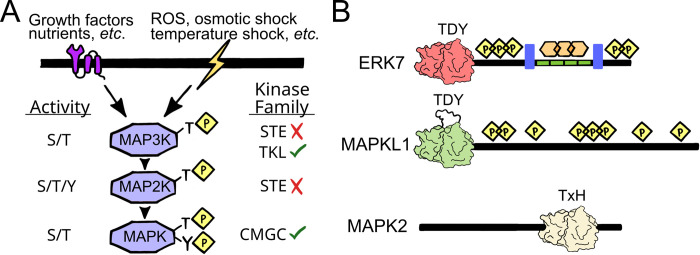
Overview of MAPK signaling. (A) In a canonical MAPK signaling cascade, a signal results in activation of the upstream MAP3K (the “MAPK kinase kinase”), which phosphorylates and activates the MAP2K (the “MAPK kinase”), which in turn phosphorylates its target MAPK on both Thr and Tyr. Apicomplexan parasites lack the STE kinase family, to which all MAP2Ks belong. They therefore encode no MAP2Ks or MAP3Ks. (B) Domain architecture of the apicomplexan MAPKs (not to scale). ERK7 and MAPKL1 both have long CTEs that are predicted to be intrinsically disordered by IUPRED [75]. The ERK7 CTE contains sequence repeats (green; Sarcocystidae only). In *Toxoplasma*, both phosphorylation (yellow diamonds) and fucosylation (orange hexagons) sites have been identified. The *Plasmodium* ERK7 CTE also has one or more predicted nuclear localization signals (purple rectangles), though no post-translational modifications of the CTE have been identified as of yet. The *Toxoplasma* MAPKL1 CTE is also phosphorylated. Many MAPKL1 family members have an extended activation loop that reaches a maximum of approximately 100 residues in Sarcocystidae. Most MAPK2 proteins have a disordered N-terminal extension. The activation motifs for each of the apicomplexan MAPK subfamilies are indicated above the kinase domain. CTE, C-terminal extension; MAPK, mitogen-activated protein kinase.

The mitogen-activated protein kinases (MAPKs), together with their regulators, comprise a core eukaryotic signaling module. The canonical MAPK module translates extracellular signals across a 3-tiered kinase cascade to provoke cellular responses (Fig 1; [1–4]). Typically, MAPKs respond to cellular stresses as well as growth and developmental cues (“mitogens”). Originally discovered in mammals and yeast, the MAPK cascades of standard model organisms have been largely well characterized. Apicomplexan parasites, however, encode atypical MAPKs whose functional networks are just beginning to be understood. Notably, apicomplexan parasites do not encode for any predicted G protein-coupled receptors or receptor kinases, which serve as the initiators of many MAPK cascades in model organisms. Apicomplexan parasites also lack STE family kinases [5–7] that are integral components of canonical MAPK cascades.

Genetic data from *Toxoplasma* have demonstrated that catalytic activity is critical to each of the 3 apicomplexan MAPK cellular functions [8–10]. These data indicate that the kinases are activated despite the lack of a canonical cascade, which begs the question: What is the mechanism of activation? Moreover, the identities of both the regulators and downstream targets of apicomplexan MAPKs remain largely unknown. Many questions remain in the biology of the apicomplexan MAPKs: What proteins do they phosphorylate to carry out their functions? What upstream signals do they respond to? By what mechanism do they become activated—by a non-standard activating kinase, an allosteric activator, or simply by scaffolding and regulatory inhibition? We also have much to learn about negative regulation of the pathways. Are apicomplexan MAPK signals tuned by phosphatases? By protein homeostasis? We will discuss these ideas as they relate to Apicomplexa in the context of the broader understanding of MAPK signaling in other organisms.

## 1) Overview of apicomplexan MAPKs

### 1.1) Clarification of apicomplexan MAPK nomenclature

Three distinct MAPKs have been identified in Apicomplexa: ERK7, MAPKL1, and MAPK2 (Fig 1B, Table 1). Two of the 3 apicomplexan MAPKs are found in *Plasmodium*. The *Plasmodium* ortholog of ERK7 was identified concurrently by 2 groups, who named it PfMAP-1/PfMRP [11,12]. The second *Plasmodium* MAPK was identified as PfMAP-2 [13]. *Toxoplasma gondii* encodes all 3 of the apicomplexan MAPKs. These are called TgMAPKL1 [8] (originally named TgMAPK1 [14]), TgMAPK2 (the ortholog of PfMAP-2, also called TgMAPK3 [15]), and TgERK7 (also called TgMAPK2 [15,16]). The lack of consistent naming conventions has led to some confusion in the field, and, for the sake of clarity, we will refer to these kinases as ERK7 (e.g., TgERK7, PfMAP-1; found in all Apicomplexa), MAPKL1 (e.g., TgMAPKL1; missing in *Plasmodium*), and MAPK2 (e.g., TgMAPK2, PfMAP-2; Alveolate-specific; found in all Apicomplexa) from here on.

**Table 1:** Apicomplexan MAPK gene models.

	ERK7	MAPKL1	MAPK2
*C*. *parvum*	cgd2_1960 (4–379)	cgd3_3030 (14–390)	cgd_4340 (130–551)
*G*. *niphandrodes*	GNI_004780 (4–413)	*Missing*	GNI_086080 (9–395)
*P*. *falciparum*	PF3D7_1431500 (14–357)	*Missing*	PF3D7_1113900 (97–491)
*T*. *gondii*	TG*_233010 (4–351)	TG*_312570 (68–548)	TG*_207820 (136–535)
*V*. *brassicaformis*	Vbra_1579 (4–348)	Vbra_18778 (42–387)	Vbra_2957 (5–409)
			Vbra_20001 (12–402)

Numbers indicate approximate boundaries of kinase domains in the sequence.

### 1.2) Evolution of MAPK signaling in Apicomplexa

Mammalian ERK7 (called “ERK8” or “MAPK15” in humans) has been described as able to robustly autophosphorylate both the Thr/Tyr of its activation loop, and thus autoactivate [17]. Such autoactivation is an unusual property of a MAPK, since the cascades are typically characterized as tightly regulated. Other members of the CMGC kinase family autophosphorylate on their activation loop Tyr [18], and ancestral reconstruction of the MAPK family supports a model where the ancestral MAPK was autoactivating [19], and traded this activity to enable more tightly responsive signaling. ERK7s are early-branching MAPKs and appear to be the most broadly conserved members of the family throughout eukaryotes [10,19]. Thus, an ERK7-like molecule is likely the original MAPK.

**Fig 2:**
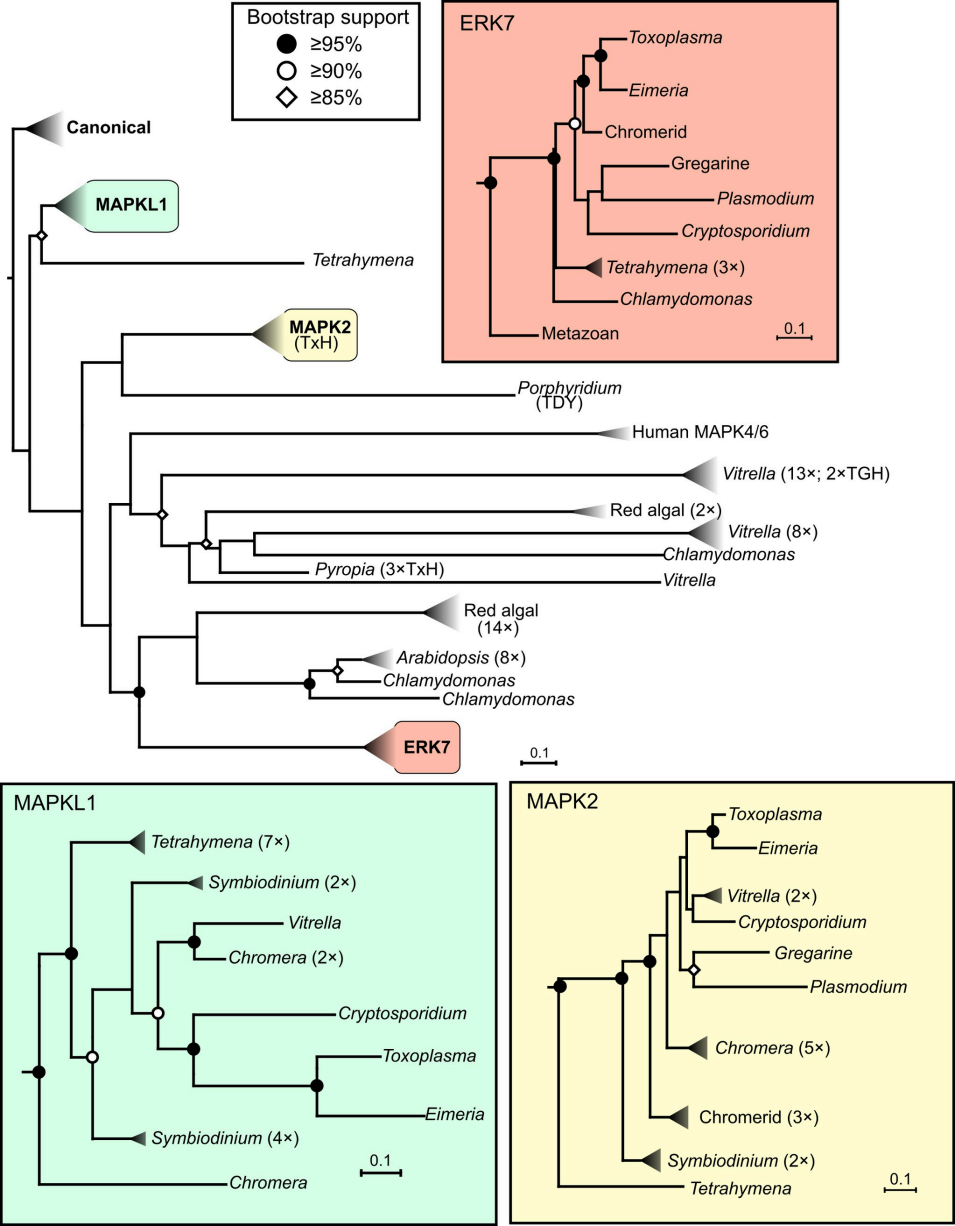
Phylogenetic analysis of the apicomplexan MAPKs. Phylogenetic trees of multiple sequence alignments of the MAPKs from the indicated organisms were estimated using IQTREE2 [76]. Subtrees were collapsed for space considerations, indicated by triangles. When present, numbers to the right of the triangles indicate number of kinases in the subtree. Expanded subtrees for the apicomplexan MAPK clades are shown in inset boxes. MAPK activation loop motifs (e.g., TDY, TxH) have been indicated, where appropriate, to demonstrate that the TxH motif is not confined to the MAPK2 clade. Organisms: Metazoan (human, mouse, fruit fly), Apicomplexa (*T*. *gondii*, *E*. *falciformis*, *E*. *maxima*, *C*. *parvum*, *G*. *niphandrodes*, *P*. *berghei*, *P*. *falciparum*, *P*. *vivax*), Chromerid (*C*. *velia*, *V*. *brassicaformis*), Dinoflagellate (*S*. *microadriaticum*), Ciliate (*T*. *thermophila*), green plant (*A*. *thaliana*, *C*. *reinhardtii*), red algae (*C*. *crispus*, *P*. *purpureum*, *P*. *yezoensis*). Note: The estimated number of MAPKs in *V*. *brassicaformis* is approximately 40 and approximately 75 in *C*. *velia;* note that analysis is complicated by current low quality of genome build and lack of verification of gene models by transcript sequencing. MAPK, mitogen-activated protein kinase.

While it is possible that ERK7 is the ancestor of all extant apicomplexan MAPKs, such a model is not supported by phylogenetic analysis (Fig 2). The number of available apicomplexan genomes has exploded in recent years and have been supplemented by the genomic sequences of closely related organisms such as those of the phylum Chromerida [20]. Notably, Chromerida, unlike Apicomplexa, appear to encode the upstream activating MAPK kinases (or “MAP2Ks”) and a much larger number of MAPKs (Fig 2), suggesting they possess a functional canonical MAPK cascade. Comparing the MAPKs across Apicomplexa with a diverse set of outgroups reveals that ERK7 from apicomplexan species branches with the orthologs from all other organisms compared (Fig 2). Furthermore, the MAPKL1 and MAPK2 clades clearly branch well outside of the ERK7 family. MAPK2 is well conserved throughout Alveolate phyla (Fig 2; [6,7,9]). There is strong support for MAPKL1 orthologs both in *Cryptosporidium* and in the Chromerids. It would therefore appear that each of the 3 apicomplexan MAPKs were found in the ancestral organism and that these proteins originally evolved in the context of a fully functional MAPK cascade.

### 1.3) Apicomplexan MAPKs are each atypical

While the apicomplexan MAPKs are divergent, phylogenetic analysis robustly supports their identification as bona fide members of the family [5] (Fig 2). Typical MAPK proteins have little more than a kinase domain and have specialized their functions by variation in the MAPK scaffolding domains ([21]; described in detail below). Each of the 3 apicomplexan MAPKs have regions apart from the kinase domain (Fig 1B). Both ERK7 and MAPKL1 have sizeable C-terminal extensions (CTEs) that are predicted to be largely intrinsically disordered, while MAPK2 contains a shorter N-terminal extension. Many MAPKL1 proteins also have an extension in the activation loop region between the DFG and APE motifs, which is most extensive (approximately 100 residues) and potentially structured in Sarcocystidae.

The ERK7 CTE is a hallmark of this MAPK subfamily and has been implicated in regulation of kinase activity and subcellular localization in the mammalian protein [22]. Notably, the TgERK7 CTE is extensively post-translationally modified, including a number of phosphorylation [23] and fucosylation sites [24], though their functional importance has yet to be investigated. Furthermore, the ERK7 CTE in *P*. *berghei* is required for its nuclear localization, due to 2 nuclear localization signals (NLSs) in the region [25]. Intriguingly, ERK7 nuclear localization is not conserved in all Apicomplexan, as the NLS are notably absent in the *Cryptosporidium* and *Toxoplasma* proteins, and TgERK7 does not localize to the parasite nucleus, at least in tachyzoites [10].

Dual phosphorylation on the activation loop Thr/Tyr is the key to MAPK regulation (Fig 1A). The MAPK2 subfamily is therefore quite unusual in that it replaced the TxY activation motif with a TxH. This indicates that the MAPK2 family must be activated in a totally distinct mechanism from typical MAPKs, even in organisms that encode the full MAPK cascade. While His is not normally considered a phosphorylatable residue in eukaryotes, this may be due to difficulty in detection. Indeed, His acts as a transition phosphoacceptor in bacterial and plant 2-component signaling [26]. Furthermore, recent advances in mass spectrometry [27,28] and development of phospho-His-specific antibodies [29] have revealed potential roles for phospho-His in metazoan biology. Finally, Apicomplexa encode for members of the nucleoside diphosphate kinase family (e.g., TGME49_295350), which has been suggested to moonlight as a His kinase in metazoa [30]. Nevertheless, it is unclear how the conserved activation loop His affects MAPK2 activation or function, and certainly remains untested whether this residue is phosphorylated in parasites. Intriguingly, some (but not all) red algae encode MAPKs with a similar TxH motif to MAPK2 [31]. It is thus tantalizing to hypothesize that the MAPK2 family evolved from horizontal gene transfer from the red algal secondary endosymbiont shared among Alveolates. Phylogenetic analysis of available sequences, however, suggests that a TxH activation motif may have evolved multiple times in the MAPKs of red algal-derived lineages (Fig 2).

## 2) Regulation of the apicomplexan MAPKs

### 2.1) Phosphorylation is the first layer of MAPK regulation

**Fig 3:**
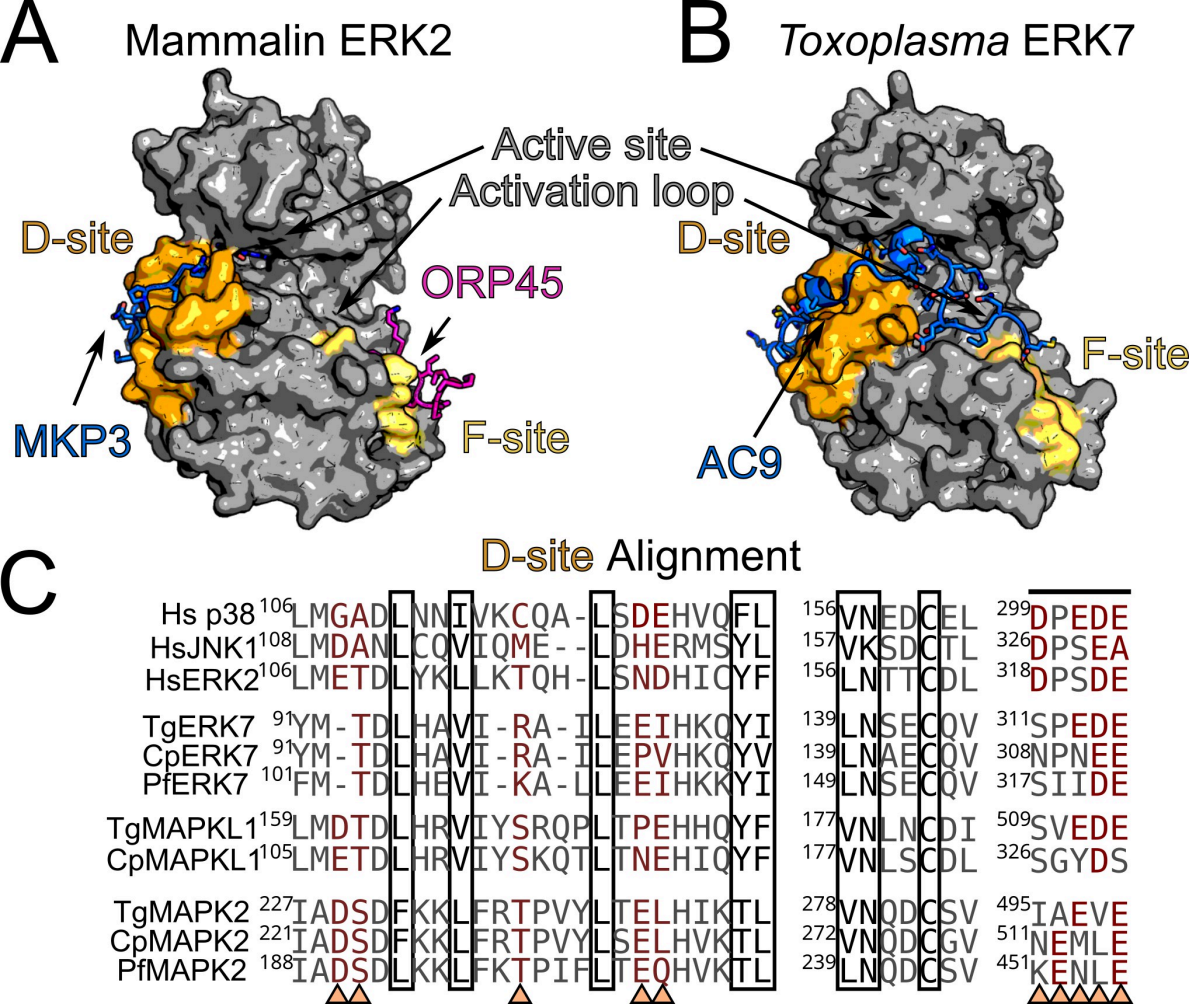
MAPKs use docking sites to recognize substrates and regulators. (A) Canonical MAPKs use the conserved D-site (orange) to recognize kinase-interaction-motifs such as that of MKP3 bound to ERK2 (blue; PDB: 2FYS). This site lies distal to the active site and substrate recognition region. Some MAPKs use a second docking site, the F-site (yellow) to recognize F-x-F-P motifs such as that found in ORP45 (purple; PDB: 7OPM). (B) The inhibitory scaffold AC9 wraps around the *Toxoplasma* ERK7 kinase domain (blue; PDB: 6V6A) and occupies both the D-site (orange), active site, and substrate-recognition region. The site where the F-site would be localized on the TgERK7 structure is indicated in yellow, though no F-site binding partners have yet been identified for an apicomplexan MAPK. It is possible that apicomplexan MAPKs do not use F-site recognition. (C) Alignment of the sequences comprising the D-site of the indicated MAPKs. Polar (mostly acidic) sites that are thought to provide specificity to the KIMs recognized are indicated by arrowheads and red text. Sites that typically make backbone or hydrophobic interactions with KIMs are boxed. (Hs–human; Tg–*T*. *gondii*; Cp–*C*. *parvum*; Pf–*P*. *falciparum*) KIM, kinase-interacting motif; MAPK, mitogen-activated protein kinase.

As described above, MAPKs are unusual in requiring phosphorylation on both a Thr and Tyr in their activation loops in order to signal, though the MAP2Ks that carry this out are missing in Apicomplexa (Fig 1A). While ERK7 family members autoactivate, and therefore do not require an upstream kinase [17,19], it unclear, however, whether the same is true for MAPKL1 or MAPK2. No interacting partners have yet been identified for a MAPKL1 protein. In *Plasmodium*, 2 NEK kinases, PfNEK1 and PfNEK3, have been demonstrated to be able to phosphorylate the PfMAPK2 activation loop in vitro, and therefore have been suggested to act as activating kinases for this protein [32,33]. However, such an activating relationship between the kinases has not been rigorously validated. It has yet to be demonstrated that PfNEK1 nor PfNEK3 are required for PfMAPK2 function within the parasites. Importantly, kinases that are *bona fide* activators of MAPKs typically contain a docking sequence motif that facilitates efficiency and specificity of activation (Fig 3; described below). Thus, identification of apicomplexan MAPKs regulators is likely to come from interactome studies.

As MAPKs require dual phosphorylation to activate, they can also be deactivated by dephosphorylation, which is carried out by specific dual-specificity phosphatases (DUSPs). DUSPs are a critical component of tuning MAPK response [34,35] and recognize their cognate MAPKs through scaffolding interactions on the D-site (described below; Fig 3). Note that kinases that robustly autoactivate, such as ERK7, cannot be primarily regulated by dephosphorylation without some additional inhibitory interaction, as the dephosphorylated kinase would quickly reactivate. MAPKL1 and MAPK2, however, may well be regulated by a more typical mechanism. We note that putative DUSP family members are encoded in all apicomplexan genomes (e.g., 9 in *Toxoplasma*, 3 in *P*. *falciparum*), though these have not yet been phenotypically or biochemically characterized. Importantly, many DUSPs regulate diverse substrates beyond MAPKs [36], so it is entirely possible these proteins regulate processes distinct from MAPK signaling in Apicomplexa.

### 2.2) Scaffolding interactions define the architecture of a MAPK cascade

Pawson and colleagues spearheaded the understanding of signaling cascades as being assembled by combinations of interactions between modular domains [37,38]. While scaffolding proteins certainly guide the architecture of canonical MAPK cascades [39–42], MAPKs, in general, recognize both their regulators and substrates using conserved docking sites on the kinase domain [43]. Docking site interactions typically have affinities in the high nanomolar to low micromolar range and have been demonstrated to be the main drivers of signaling specificity [21]. Therefore, defining such docking interactions for the apicomplexan MAPKs is potentially the most direct method by which we can identify potential activators and substrates and thus delineate their signaling pathways.

Two distinct docking sites have been identified on the MAPK kinase domain. The first, called the D-site or common docking (CD) domain, is thought to be present in all MAPKs. The D-site lies on the face opposite of the kinase active site and binds so-called “kinase-interacting motifs” or KIMs [43–46] (Fig 3). Most often, KIMs are short linear sequence motifs containing an N-terminal basic patch followed by a hydrophobic patch (e.g., [K/R]-X-[K/R]-X_2-4_-[I/L]-X-[I/L]) and are unfolded until binding [47,48]. Some interacting proteins, however, such as the dual-specificity phosphatases that down-regulate canonical MAPKs, bind the D-site using a folded surface [49]. The second site, called the F-site or DEF-site, lies on the C-lobe just below the activation loop [47,50] and typically recognizes short linear motifs containing the sequence F-X-F-P [51] (Fig 3). While the D-site appears conserved in all MAPKs, the F-site binding seems to be missing in some MAPKs and has therefore been proposed to provide an additional layer of specificity to substrate recognition [51–53]. Even though the motifs recognized by the 2 docking sites are relatively relaxed, there is a surprising degree of functional specificity in practice, suggesting there is still much to learn about the determinants of recognition. We note that the primary sequence and structural elements of both docking sites appear intact in each of the 3 apicomplexan MAPKs, suggesting that they recognize similar motifs those found in metazoan proteins.

### 2.3) Regulation by inhibitory interactions

In addition to regulation by phosphorylation, MAPKs, like other kinases, may be inactivated by binding inhibitor proteins. The scaffolding protein PEA-15 sequesters metazoan ERK2 in the cytoplasm [54]. PEA-15 binds near the ERK2 F-site, which results in conformational changes to the activation loop, the Gly-rich loop, and the α-C helix, inactivating the kinase [53]. A number of pathogens, such as KSHV and *Yersinia*, use proteins with optimized docking site motifs to compete off the endogenous partners of kinases such as mammalian RSK and ERK2 [50,55], thereby blocking kinase function.

Such a mechanism of regulation is likely especially important for kinases such as ERK7 that are autoactivating. Indeed, we have found that that an inhibitory scaffold is essential to ERK7 function in *Toxoplasma*. We demonstrated that *Toxoplasma* apical cap protein 9 (AC9) binds tightly to the ERK7 kinase domain, and is required for its recruitment to the parasite apical cap, and thus for its essential function [56]. AC9 interacts with TgERK7 with a surprisingly large interface, wrapping around the kinase to occupy the D-site docking domain, the ATP binding site, and the substrate recognition site of the kinase [56] (Fig 3 –docking domain). While the net binding affinity of AC9 for ERK7 is relatively high (K_D_ approximately 50 nM), the distribution of the binding energy over such a large surface leads to an interaction that is relatively dynamic. This led to a model whereby AC9 acts both to concentrate TgERK7 at its site of action and to block phosphorylation of nonspecific substrates. AC9 can be effectively competed off by substrates containing motifs that recognize the D-site with only moderate affinity (1 to 10 μM) [56]. In fact, we recently found that another apical cap protein, AC10, is one such substrate of ERK7 [57]. Furthermore, it appears that AC9 and AC10 use multivalent interactions to form an amorphous oligomer at the apical cap IMC to concentrate TgERK7 at this site [57] and facilitate its function.

### 2.4) Regulation by protein homeostasis

Another major mechanism of kinase regulation is, of course, protein homeostasis. Each of the 3 *Toxoplasma* MAPKs show strong cell cycle dependence of their transcript levels (see EupathDB; [58,59]). All 3 *Toxoplasma* MAPKs have transcript levels that vary with the tachyzoite cell cycle (ToxoDB v53). While TgERK7 protein is found throughout the cell cycle [10], TgMAPKL1 is found only during S-phase and mitosis [60]. TgMAPK2 protein levels also appear cell cycle regulated, and the protein is undetectable from late budding through cytokinesis [9]. Notably, the TgMAPK2-AID phenotype was not completely rescued by expression of a non-degradable (i.e., not AID-tagged) copy of the protein [9]. Thus, a careful balance of protein expression and degradation may be critical to the tuning of apicomplexan MAPK signaling.

## 3) Biological functions associated with the apicomplexan MAPKs

**Fig 4:**
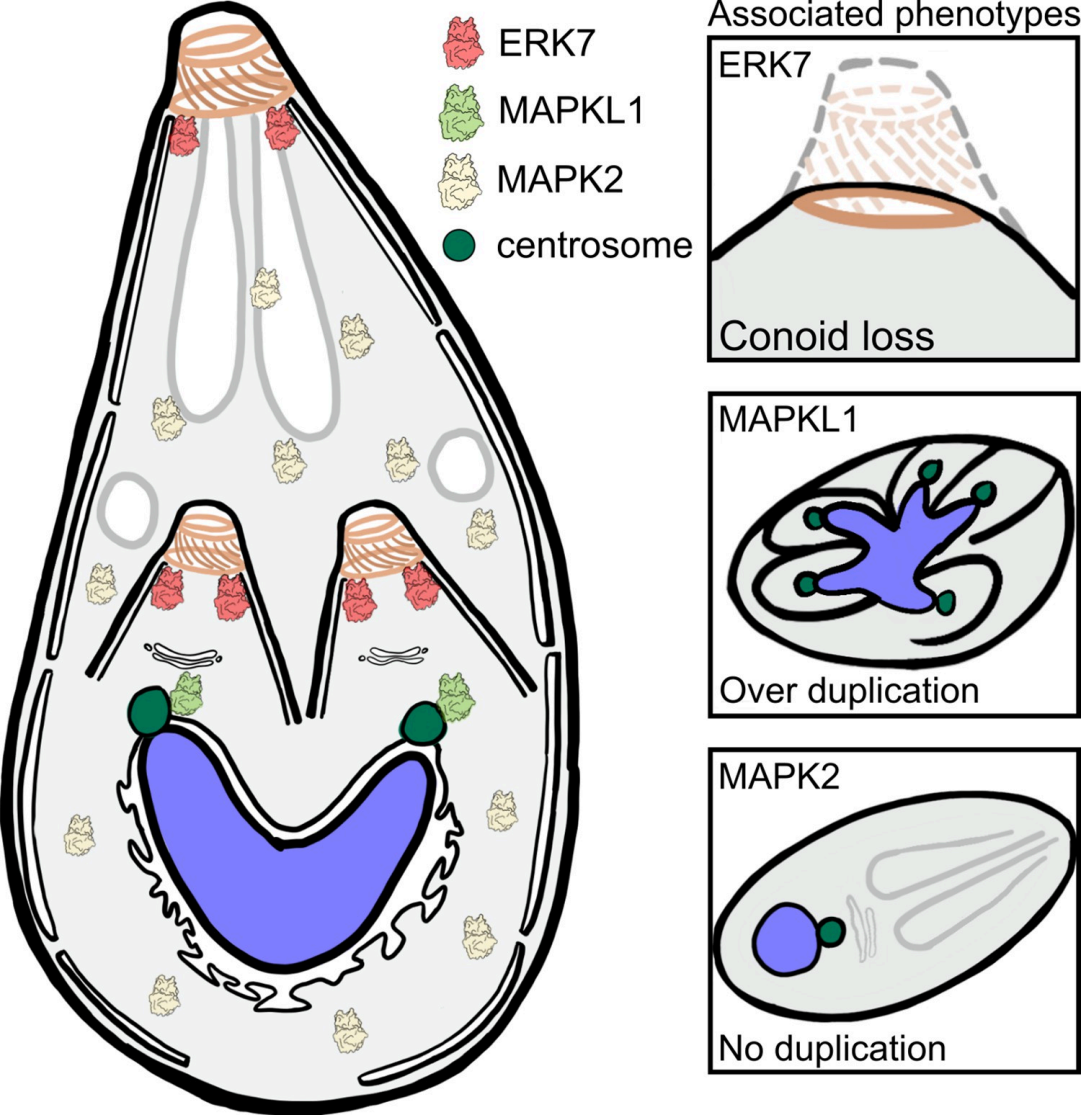
MAPK function in *Toxoplasma* tachyzoite replication. ERK7 (red kinase) localizes to the maternal and daughter bud apical caps, just below the conoid (orange rings). MAPKL1 (green kinase) localizes to the pericentrosomal material surrounding the centrosome (green circle). MAPK2 (yellow kinase) is cytosolic. Right panels show phenotypes resulting from knockdown of each of MAPKs. Loss of ERK7 results in destruction of the conoid. Loss of MAPKL1 and MAPK2 has opposing effects on centrosome duplication. MAPK, mitogen-activated protein kinase.

Recent work has defined functions for each of the apicomplexan MAPKs (Fig 4). As the majority of work has been conducted in *Toxoplasma* and *Plasmodium*, we will focus on the current understanding of MAPK function in those organisms.

### 3.1) MAPKL1

TgMAPKL1 localizes to the outer core of the centrosome of dividing *Toxoplasma* tachyzoites, and use of a temperature-sensitive allele demonstrated that its loss-of-function results in over-duplication of centrosomes [60]. This led to the model that nuclear and cellular division are controlled separately in *Toxoplasma* and related organisms [60]; MAPKL1 and other outer core centrosome components are proposed to control parasite budding, while inner core components control nuclear division. Given its tight association with the centrosome, MAPKL1 likely has multiple tight interacting partners, though no interactome study for this kinase has yet been published.

Notably, MAPKL1 has been demonstrated to be susceptible to inhibition by at least 2 available compounds that are marketed as selective. The ALK4,5,7 inhibitor SB505124 was demonstrated to block parasite replication both by inhibiting MAPKL1 and by altering host HIF1α signaling [8]. MAPKL1 also has a relatively small “gatekeeper” residue (Ser191) that renders it susceptible to inhibition by bumped inhibitors such as 1NM-PP1 [61]. Notably, TgCDPK1 is also potently inhibited by bumped inhibitors, blocking parasite motility and invasion [62]. The block on cell cycle could be rescued, however, by mutating the MAPKL1 gatekeeper to a more typical, bulky Tyr, verifying MAPKL1 as the relevant target [61]. While these studies highlight the potential (and mostly untapped) value of pharmacological inhibition to study biological function of apicomplexan kinases, they also demonstrate the importance of verifying specificity of a given drug when using it a new system (i.e., most parasites), and thus serve as important cautionary tales.

### 3.2) MAPK2

In P*lasmodium*, MAPK2 was found to be essential for male gametogenesis and ex-flagellation, a process by which the male gametocytes undergo 3 rounds of replication leading to the production of 4 flagellated gametes [63,64]. While MAPK2 was demonstrated dispensable in *P*. *berghei* asexual stages, early attempts to knock out the kinase in *P*. *falciparum* in the blood stages [65] led to the hypothesis that MAPK2 may have different functions in the 2 species in the asexual stages. However, a recent, unconditional, knockout of PfMAPK2 demonstrates that MAPK2 is dispensable in the asexual stages of both species [63,64]. Notably, while *Plasmodium* MAPK2 is primarily nuclear-localized in gametocytes [63], expression of an exogenous copy in the blood stages yields primarily cytosolic localization [65].

Somewhat surprisingly, given its function in *Plasmodium* and conservation throughout Alveolates, MAPK2 is essential in the asexual tachyzoite stage of *Toxoplasma* [9], though its role in the sexual stages has yet to be tested. When TgMAPK2 was conditionally depleted using the auxin-inducible degron system, parasite replication was arrested prior to the initiation of centrosome duplication and daughter cell budding [9]. This appeared to be a true arrest, as wash-out of auxin (and restoration of TgMAPK2 protein), rescued the phenotype in the majority of parasites. Remarkably, both the parasite cell and its organelles continued to grow in size without segregation of contents into buds or parasite division [9]. Thus, TgMAPK2 is required for maintaining the coupling of cell growth with division.

Intriguingly, MAPKL1 and MAPK2 loss-of-function in *Toxoplasma* both manifest in opposing phenotypes associated with centrosome duplication. While the loss of TgMAPKL1 results in over-duplication of the centrosome, loss of TgMAPK2 blocks duplication. Interestingly, while TgMAPKL1 is tightly associated with the centrosome [60], TgMAPK2 is broadly cytosolic and does not co-localize with any known cellular structures, including the centrosome [9]. Thus, TgMAPK2 likely facilitates centrosome duplication more indirectly than does TgMAPKL1. As with MAPKL1, there are, as of yet, no validated interactors for MAPK2 in any organism, so both its upstream regulators and downstream targets are a mystery. Notably, MAPKs often regulate transcription factor localization and activity, including the AP2 transcription factors in plants [66,67], which are also conserved in Apicomplexa [68,69]. One possibility that deserves consideration is that kinases, such as MAPK2, play a similar role in Apicomplexa.

### 3.3) ERK7

Compared to metazoa, where it is one of the most poorly understood MAPKs, in Apicomplexa, the best understood MAPK pathway is that of *Toxoplasma* ERK7, which is essential to the tachyzoite lytic cycle [10]. TgERK7 localizes to the apical cap of the parasite inner membrane complex (IMC), both in daughter buds and in mature parasites [10], though its staining becomes weaker in the mature parasites as they approach cytokinesis. When TgERK7 was inducibly knocked-down with the auxin-degron system, *Toxoplasma* tachyzoites replicated normally within a vacuole, but were immotile and therefore incapable of egress and invasion of new host cells [10]. In parasites without ERK7, these phenotypes are all due to the loss of the parasite conoid, the central organizing hub of the apical complex. As the conoid was preserved in early daughter buds, but missing in mature parasites grown without ERK7, the kinase was posited to play a role late in conoid assembly [10]. Recent findings have called into question this mechanism of action.

We recently completed an interactome study of TgERK7 [70]. In addition to its known regulatory scaffolds AC9 [56] and AC10 [57], we identified a putative E3 ligase called CSAR1 that directly interacted with the ERK7 by yeast-2-hybrid. Remarkably, knockout of CSAR1 suppressed the ERK7 loss-of-function phenotype, allowing parasites to mature with intact conoids. Thus, it appears loss-of-function of ERK7 leads to an aberrant function in CSAR1, causing the premature degradation of daughter conoids [70]. This idea is consistent with recent proteomics that indicated loss of ERK7 results in degradation of components of the apical complex [71]. Intriguingly, ERK7 has been strongly linked both to the biogenesis and maintenance of primary cilia in metazoa [72,73] as well as to regulation of ubiquitin-mediated degradation of specific proteins [74]. It seems likely, therefore, that different apicomplexan parasites have adapted conserved ERK7 functions to their varied life cycles.

In stark contrast to findings in *Toxoplasma*, the *Plasmodium falciparum* ERK7 ortholog (PfMAP-1) is apparently dispensable both for the blood stage and mosquito stages of the parasite’s life cycle [65]. What the kinase is doing in these parasites is therefore unknown. *Plasmodium* ERK7 localization is dynamic as parasites develop: *P*. *berghei* ERK7 was found to be enriched in the parasite nuclei in early liver stage schizonts. In the cytomere stage, PbERK7 was instead concentrated to comma- and ring-shaped structures that no longer co-localized with the nucleus, while in merozoites ERK7 displayed uniform cytosolic localization [25]. It is unfortunate that PbERK7 localization has not yet been experimentally compared with any additional marker, such as the IMC. Notably, PbERK7 expression is much higher in parasite stages such as ookinetes and sporozoites, suggesting its localization and function may be distinct in these stages.

## Concluding remarks

Much of the fungal and metazoan MAPK signaling networks were elucidated by a combination of genetic screens, early systems biology methods (e.g., yeast-2-hybrid), and painstaking biochemistry. Identifying the full complement of activators, negative regulators, scaffolds, and downstream substrates of the apicomplexan MAPKs will take similar efforts. Importantly, the depth of understanding of human MAPK signaling has led to >10 FDA-approved small molecules to date (with more in clinical trials) and a plethora of tool compounds that target these pathways. Kinase signaling therefore represents an, as yet, untapped bounty of targets to treat apicomplexan infections. Of course, modern proteomics and bioinformatics methods can greatly accelerate discovery. However, to gain the depth of knowledge required to unravel the complexity of parasite kinase signaling will require the same kind of careful, hypothesis-driven cell biological, and biochemical work that enabled our current understanding in well-studied models.
